# Plasma phospholipids profiling changes were associated with the therapeutic response to Roxadustat in peritoneal dialysis patients

**DOI:** 10.3389/fphys.2023.1279578

**Published:** 2023-12-18

**Authors:** Ya-Hui Yang, Yishakejiang Saimaiti, Yang Zhao, Wen Tang

**Affiliations:** ^1^ Department of Nephrology, Peking University Third Hospital, Beijing, China; ^2^ Department of Laboratory Medicine, Peking University Third Hospital, Beijing, China

**Keywords:** peritoneal dialysis, phospholipids, hypoxia inducible factor prolyl hydroxylase inhibitors, anemia, end-stage kidney disease

## Abstract

**Background:** Elevated Phospholipids (PLs) and sphingolipid (SM) metabolism relates to with poor clinical status and adverse outcome of end-stage kidney disease patients undergoing peritoneal dialysis (PD). Studies have suggested that the use of hypoxia-inducible factor prolyl hydroxylase inhibitor (HIF-PHI) (Roxadustat) is associated with altered lipid metabolism. Observing on how PLs and SMs changes after the HIF-PHI treatment in PD patients may help understand the possible effect of HIF-PHI on PD patients besides correcting of anemia.

**Materials and methods:** Stable peritoneal dialysis (PD) patients treated with Roxadustat for over 3 months were included. Phospholipid and sphingolipid metabolism were measured before and after treatment.

**Results:** 25 PD patients were included. Overall, phospholipid and sphingolipid metabolism showed a decreasing trend after HIF-PHI treatment. Levels of LysoPC (20:0), 1,2-dilinoleoyl-sn-glycero-3-phosphocholine [CisPC (DLPC) (18:2)], lysophosphatidylethanolamine (LysoPE) (14:0), and sphingomyelin (d18:1/17:0) (17:0) were significantly decreased (all *p* < 0.05). Further regression analyses confirmed the significant relationship between the increased of hemoglobin levels and the decrease in egg lyso PC: phosphatidylethanolamines (PE) (16:0–18:1), PE (16:0–18:2), PE (16:0–22:6), PE (18:0–20:4), PE (18:0–18:2), LysoPE (18:0), LysoPE (18:1), and phosphatidylcholine (PC) (18:1–18:0).

**Conclusion:** This study demonstrated that phospholipid and sphingolipid metabolism decreased after administration of HIF-PHI and was associated with improvement of anemia.

## Introduction

Roxadustat, a hypoxia-inducible factor prolyl hydroxylase inhibitor (HIF-PHI) is a novel small-molecule oral drug used for the treatment of renal anemia (Sanghani and Haase, 2019) that targets all three hypoxia-inducible factor prolyl hydroxylase domain (HIF-PHDs) to a similar extent (Yeh, 2017). Small-molecule prolyl hydroxylase domain (PHD) inhibitors (PHDIs) stabilize HIF by inhibiting PHD, thereby promoting the secretion of erythropoietin (EPO) to promote hematopoiesis (Rabinowitz, 2013; Duan, 2016).

In addition to the treatment of anemia, several clinical studies have suggested that Roxadustat treatment is accompanied by a reduction in blood lipid levels. Two clinical trials have shown that Roxadustat decreases total, low density lipoprotein (LDL), and high density lipoprotein (HDL) cholesterol levels in patients with non-dialysis-dependent chronic kidney disease (Provenzano, 2016; Chen H., 2017a). Moreover, Chen reported a decrease in triglycerides and very low density lipoprotein (VLDL)-cholesterol in Roxadustat-treated patients with chronic kidney disease (Chen N., 2017b). Interestingly, Zhang showed that the adipocyte HIF-2α reduces atherosclerosis by promoting ceramide catabolism, thus increasing hepatic cholesterol elimination and thermogenesis (Zhang). Atherosclerosis amelioration can be pharmacologically achieved in mice by activating adipose HIF-2α via FG-4592 (Roxadustat).

Sphingolipid metabolites, particularly ceramide and sphingosine-1-phosphate, are signaling molecules that regulate a diverse range of cellular processes that are important in immunity and inflammatory disorders (Maceyka and Spiegel, 2014; Papazoglou, 2022). Phospholipids (PLs) are integral components of the membrane and have important functional, structural, and metabolic roles. Recent reports advocate the involvement of PLs in signaling pathways that modulate pathophysiological disease states, including inflammation, oxidative stress, angiogenesis, and apoptosis. Phosphatidylcholines (PCs) have been proven to independently predict the occurrence of future cardiovascular (CV) events in patients with established CAD (Hilvo, 2020). Moreover, in peritoneal dialysis (PD) patients, our previous study demonstrated that poor volume status was associated with the changes of PE and PC (Li, 2013). In addition, we have found that PD technical failure patients had different plasma PL profiles to non-failure patients using a lipidomic method, predominantly of SM and PC PLs (Tang, 2014a).

Therefore, observation on how PLs and SMs changes after the HIF-PHI treatment in PD patients might help to understand the possible effect of HIF-PHI on PD patients besides correcting of anemia. Novel insights into lipid metabolism could elucidate its mechanism of action and effects on possible PD patients outcome. Thus, in the present study, both serum lipidomics and metabolomics data were analyzed after the clinical use of HIF-PHI for renal anemia treatment in patients with end-stage renal disease who underwent PD.

## Methods

### Study procedures

This was a single-center retrospective self-controlled observational study. Patients’ blood sample were prospectively collected for routine evaluation while patients were retrospectively included according to the inclusion criteria and the blood were tested accordingly. Patients undergoing stable peritoneal dialysis visited our clinic for clinical evaluation, and blood tests were performed between December 2019 and December 2021. They were included according to whether they used HIF-PHIs (Roxadustat) for more than 3 months during the study period. The exclusion criteria were the 1) Discontinuation of HIF-PHI for various reasons during the study period. 2) Severe heart failure [New York Heart Association (NYHA) grade III or above], liver cirrhosis, autoimmune disease, and tumor; 3) Acute complications, such as infection, volume overload, surgery, cardiovascular or cerebrovascular diseases, and blood transfusion due to anemia during Roxadustat treatment; 4) Significant changes in the dialysis dose and the dialysis regimen; 5) The doses of various lipid-decreasing drugs and sevelamer adjusted during the HIF-PHI treatment; and 6) Blood samples before and after Roxadustat treatment were unavailable.

Clinical data, demographic data, laboratory examinations, and use of HIF-PHIs were collected from the available medical records. Biochemical assays and complete blood counts were performed.

### Serum lipidomics analysis and metabolomics data processing

Sample pretreatment: Study samples, preserved at −80°C, were thawed at 4°C. Each sample (10 μL) was added, followed by an internal standard solution (10 μL), 0.9% NaCl (10 μL), and chloroform:methanol (2:1) (100 μL). The mixture was vortexed for 20 s and allowed to stand for 30 min at 4°C. After centrifugation for 3 min at 7,800 g, 20 μL of the supernatant was transferred into the insert and concentrated to dryness under nitrogen. Before injection for liquid chromatography-tandem mass spectrometry (LC-MS/MS) analysis, the dried samples were re-dissolved in acetonitrile:isopropanol (1:1) (20 μL) and vortexed for 60 s.

### LC-MS/MS analysis

The lysophosphatidylcholine (LysoPC) components were identified and quantified using Eksigentultral liquid chromatography 100 coupled with an AB 5600 Triple TOF system (AB SCIEX) and separated using a 2.1 mm × 100 mm XBridge Peptide BEH C18 column (Waters) with a 4 mm × 2.0 mm guard column (Phenomenex). The separation of LysoPC was achieved at a column temperature of 40°C using acetic acid amine (10 mM), formic acid (0.1%, v/v), and water (99.9%, v/v) as mobile phase A, and acetic acid amine (10 mM), formic acid (0.1%, v/v), acetonitrile (49.95%, v/v), and isopropanol (49.95%, v/v) as mobile phase B. The step gradient was as follows: 0.01 min, 35% (v/v) B; 0.01–2 min, 35%–80% (v/v) B; 2–9 min, 80%–100% (v/v) B; 9–15 min, 100% (v/v) B; 15–16 min, 100%–35% (v/v) B; 16–20 min, 35% (v/v) B. The injection volume was 2 μL and the total run-time was 20 min at a flow rate of 0.4 mL/min. The instrument, under the negative model, was set as follows: curtain gas, ion source gas 1, and ion source gas 2 at 30, 50, and 50 psi, respectively; source temperature at 550°C; and ion spray voltage floating at −4,500 V. In the auto MS/MS acquisition, the m/z range for Time of Flight Mass Spectrometer (TOF MS) scan and production of ion scan was 100–1,200 Da and 50–1,200 Da, respectively. The collision energy of the production ion scan was set to −35 ± 15 V and the declustering potential was set to −80 V.

### Data processing

Peak View 1.2 was used to identify LysoPC, and Multi Quant 2.1 was used to quantify LysoPC based on the m/z value and sample retention time.

### Statistical analysis

All statistical analyses in this study were performed using SPSS 26.0. The frequency and percentage were used to describe categorical variables. Categorical results were analyzed using chi-square tests and presented as frequencies and percentages. The mean and standard deviation was used to describe normal distribution continuous variables and unnormal distribution continuous variables were presented as median (25th–75th percentile). The Kolmogorov-Smirnov (K-S) test was used to observe the normality of the two groups of data before and after Roxadustat treatment. In the comparative analysis of before and after Roxadustat administration and the correlation analysis between hemoglobin changes and the lipid metabolite changes, if the K-S test results of both before and after Roxadustat administration obeyed the normal distribution, the paired T-test and Pearson correlation analysis would be used for processing, respectively; if either before or after the Roxadustat groups did not obey the normal distribution, Wilcoxon signed rank test and Spearman correlation analysis were used, respectively. Linear regression analysis was used to observe whether the changes in hemoglobin were significantly correlated with changes in lipid metabolites after excluding the effects of factors, such as age, sex, total kt/v change, total ccr change, and BMI change. Metabolism levels were analyzed using MetaboAnalyst 5.0.

## Results

**FIGURE 1:**
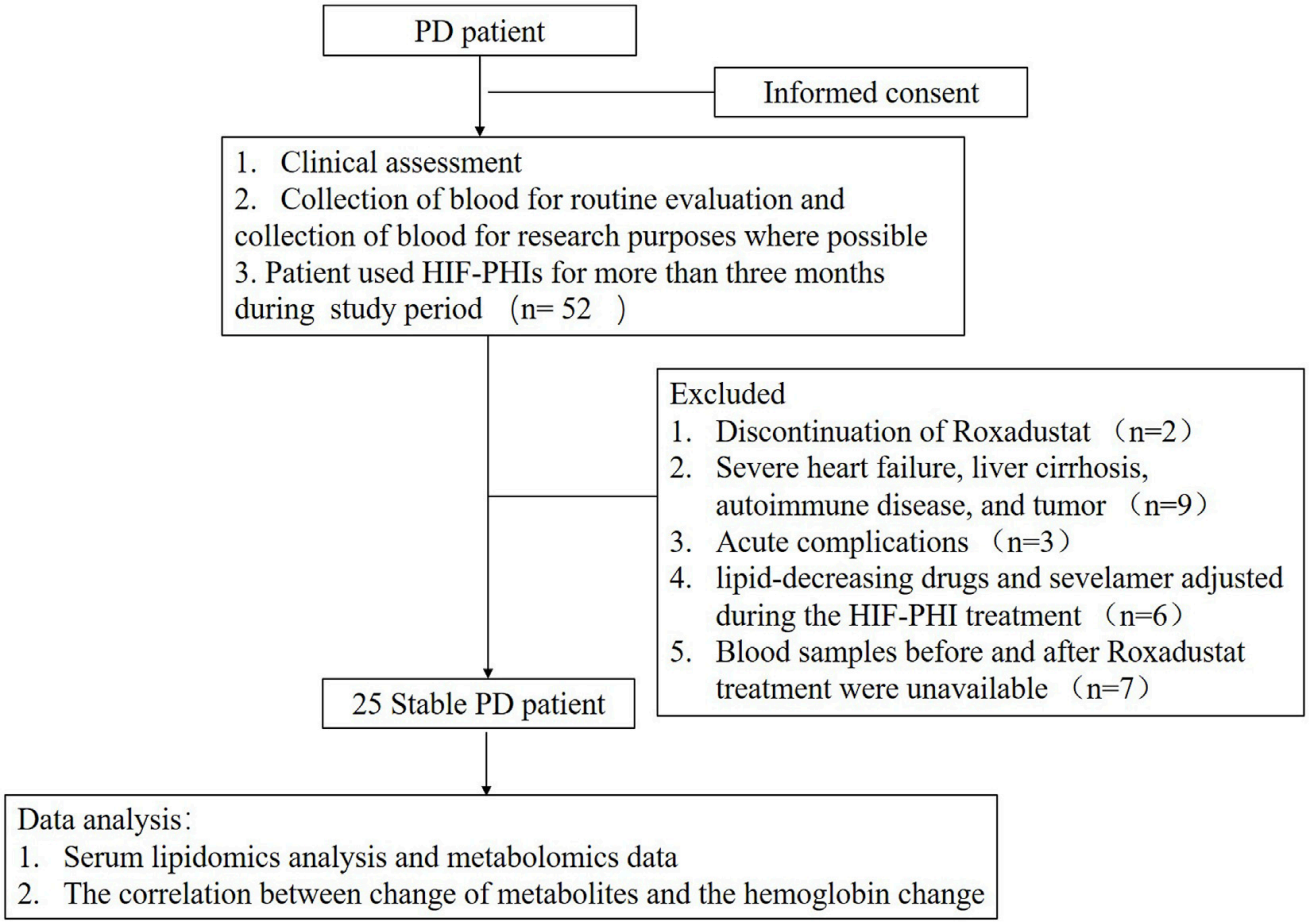
STARD diagram depicting the study design and classification of study participants.

A total of 52 patients were initially enrolled for this study in which twenty-seven patients were excluded due to different reasons and twenty-five peritoneal dialysis (PD) patients were finally included in this study and analyzed (Figure 1). The time between two serum lipidomics analyses was 29 weeks range from 20 to 44 weeks. As shown in Table 1. After Roxadustat treatment, the patient showed a significant improvement in hemoglobin levels. (*p* = 0.006). Plasma cholesterol levels, including total cholesterol, low-density cholesterol, and high-density cholesterol levels, decreased significantly. The patients’ dialysis clearance, as shown by the Kt/V and Ccr, remained stable.

**TABLE 1:** Baseline and follow-up characteristics of study population after Roxadustat treatment.

	Baseline	Follow-up	*p* value
Age (years)	57.91 ± 12.42		
BMI (kg/m^2^)	21.64 ± 2.01	21.80 ± 2.53	0.460
Hemoglobin (g/L)	106.00 ± 14.80	115.72 ± 15.02	0.006
RBC count (10^12^/L)	3.45 ± 0.57	3.73 ± 0.57	0.01
Hematocrit (%)	0.32 ± 0.05	0.36 ± 0.05	0.043
Kt/V Total	1.99 (1.83∼2.14)	1.93 (1.63∼2.07)	0.086
Ccr Total (ml/min)	73.86 (60.23∼88.32)	77.31 (60.74∼89.18)	0.67
Creatinine (μmol/L)	851.68 ± 253.47	827.36 ± 228.57	0.09
Urea (mmol/L)	19.00 ± 5.17	18.93 ± 5.35	0.93
TCHO (mmol/L)	4.93 (4.17∼5.80)	4.28 ± 1.07	0.008
Triglyceride (mmol/L)	1.60 (1.33∼3.04)	1.41 (0.89∼2.82)	0.15
LDL-C (mmol/L)	2.27 (1.94∼3.05)	2.07 (0.67∼3.05)	0.017
HDL-C (mmol/L)	1.12 ± 0.26	0.99 ± 0.29	0.007
Plasma Albumin (g/L)	36.88 ± 4.79	36.22 ± 5.34	0.268
hsCRP (mg/L)	3.08 (0.59,5.36)	1.58 (1.03,6.38)	0.468

BMI, body mass index; RBC, red blood cell; TCHO, total cholesterol; LDL-C, low density lipoprotein cholesterol; HDL-C, high density lipoprotein cholesterol; hsCRP, hypersensitive C-reactive protein.

### Metabolism levels at baseline and follow-up

**FIGURE 2:**
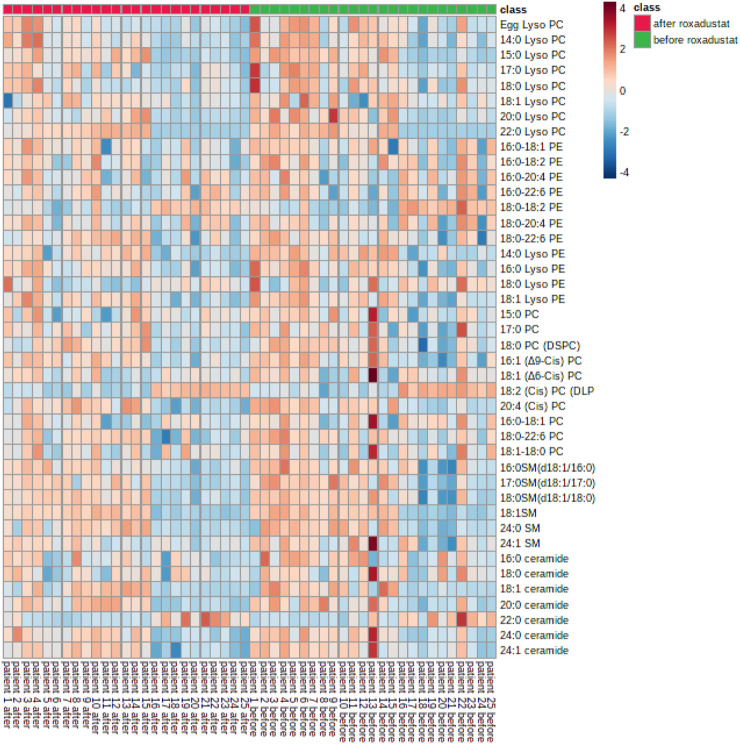
Heatmap of phospholipids and sphingolipids metabolism changes before and after Roxadustat treatment.

**FIGURE 3:**
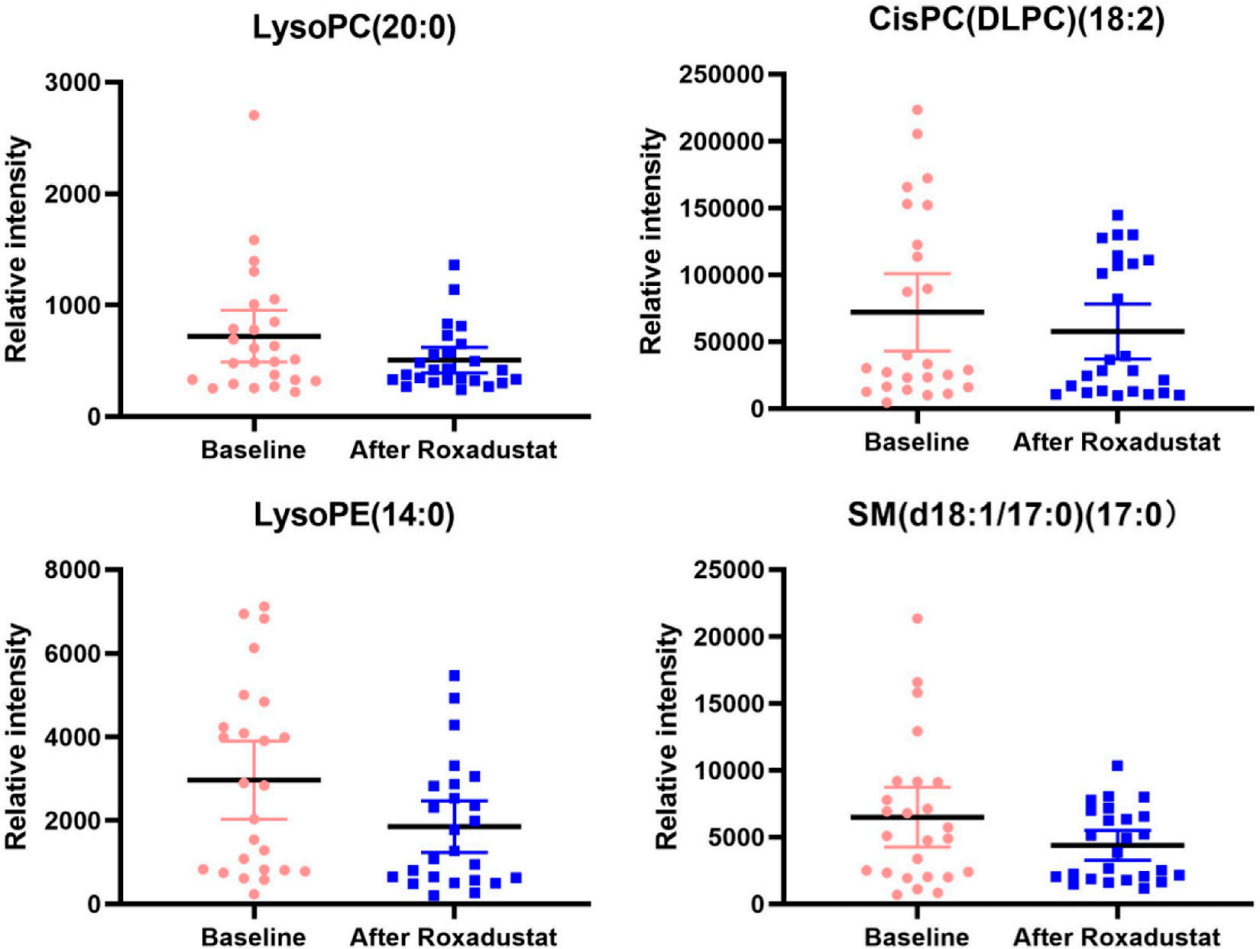
This figure presents the boxplots of the significantly changed metabolite levels at baseline and at follow-up after treatment with Roxadustat. The sample means are indicated by the black bars.

As shown in Figure 2. In general, all phospholipid and sphingolipid metabolism showed a decreasing trend after the HIF-PHI (Roxadustat) treatment. Levels of LysoPC (20:0), CisPC (DLPC) (18:2), LysoPE (14:0), and SM (d18:1/17:0) (17:0) were significantly decreased (all *p* < 0.05) after the HIF-PHI (Roxadustat) treatment (Figure 3).

### Associations between changes in metabolisms and hemoglobin change from the baseline

**FIGURE 4:**
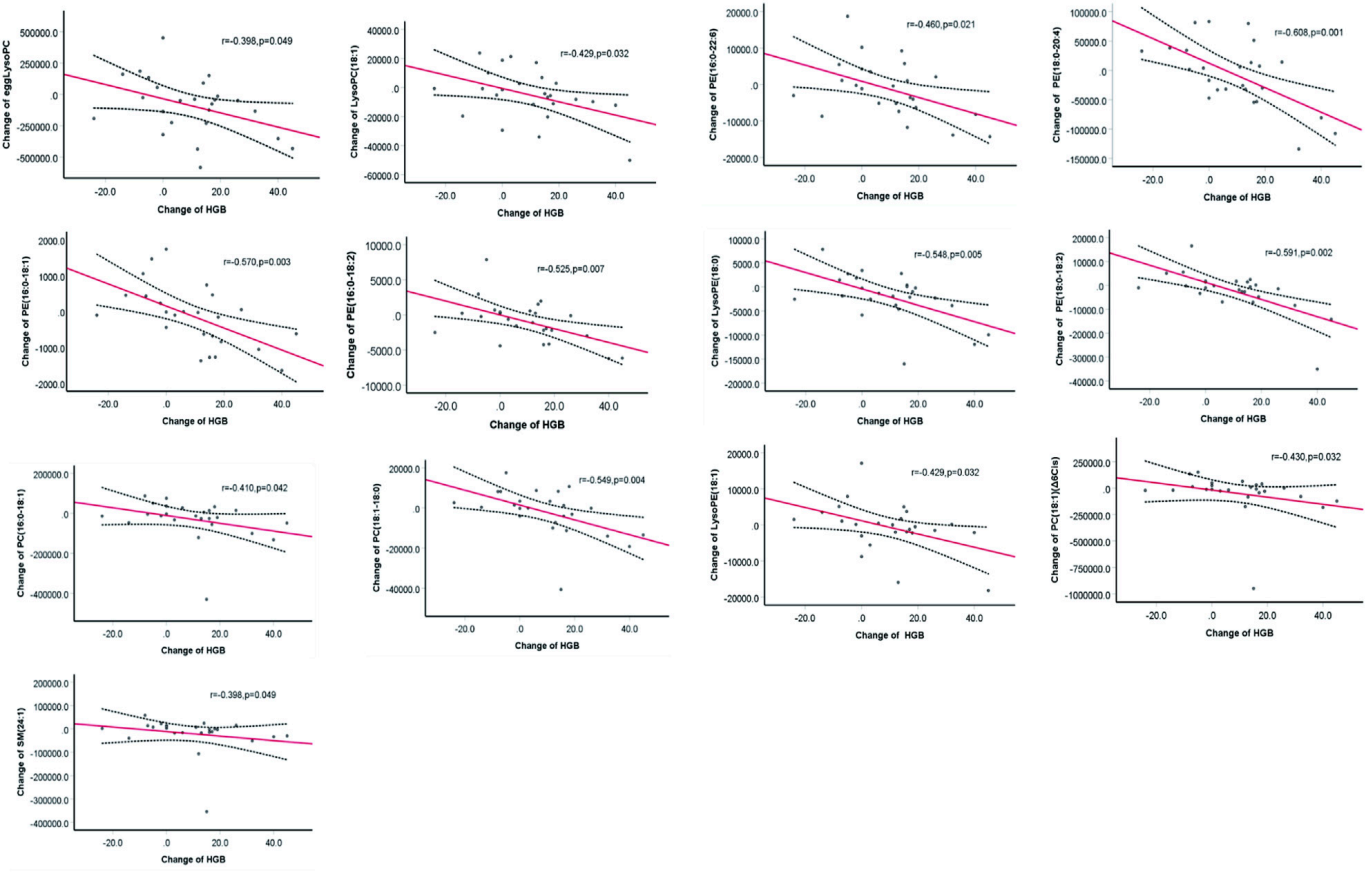
The correlation between change of metabolites and the hemoglobin change.

Reductions in the levels of eggLysoPC, LysoPC (18:1), PC (18:1) (Δ6Cis), PC (16:0–18:1), PC (18:1–18:0), PE (16:0–18:1), PE (16:0–18:2), PE (16:0–22:6), PE (18:0–20:4), LysoPE (18:0), PE (18:0–18:2), and LysoPE (18:1) SM (24:1) were negatively (*p* < 0.05) associated with the increase in hemoglobin (Figure 4). Further regression analyses confirmed the significant relationship between the increase of hemoglobin level and the decrease of egg lyso PC; PE (16:0–18:1); PE (16:0–18:2); PE (16:0–22:6); PE (18:0–20:4); PE (18:0–18:2); Lyso PE (18:0); LysoPE (18:1) and PC (18:1–18:0) after controlling for age, gender, total Kt/V change, total Ccr change, BMI change, plasma albumin change and high sensitive C- reactive protein change (Table 2).

**TABLE 2:** Multiple regression analysis between change of metabolism levels and the hemoglobin change.

Phospholipid change	HGB change			
	*β*	*t*	R^2^	*p*
Change eggLysoPC	−0.685	−3.156	0.486	0.006
Change LysoPC (18:1)	−0.479	−1.784	0.213	0.093
Change PE (16:0–18:1)	−0.671	−3.347	0.561	0.004
Change PE (16:0–18:2)	−0.386	−2.104	0.634	0.052
Change PE (16:0–22:6)	−0.379	−1.79	0.51	0.092
Change PE (18:0–20:4)	−0.598	−3.215	0.623	0.005
Change LysoPE (18:0)	−0.636	−2.976	0.502	0.009
Change PE (18:0–18:2)	−0.594	−3.319	0.651	0.004
Change LysoPE (18:1)	−0.721	−3.439	0.52	0.003
Change PC (18:1)(Δ6Cis)	−0.385	−1.465	0.246	0.162
Change PC (16:0–18:1)	−0.422	−1.587	0.229	0.132
Change PC (18:1–18:0)	−0.590	−2.624	0.448	0.018
Change SM (24:1)	−0.330	−1.213	0.19	0.243

All the initial model included age, gender, total Kt/V change, total Ccr change, BMI change, plasma albumin change and hs-CRP change.

## Discussion

The present study found that patients with end-stage kidney disease (ESKD) who underwent PD showed decreased concentrations of LysoPC (20:0), LysoPE (14:0), PC (18:2) cis dlpc, and SM (17:0) d18:1/18:0 after Roxadustat treatment. In addition, decreased levels of LysoPC, LysoPC (18:1), PC (18:1) (Δ6Cis), PC (16:0–18:1), PC (18:1–18:0), PE (16:0–18:1), PE (16:0–18:2), PE (16:0–22:6), PE (18:0–20:4), LysoPE (18:0), PE (18:0–18:2), LysoPE (18:1), and SM (24:1) were correlated with improvement from anemia. To our knowledge, this is the first study to investigate the changes in phospholipid and sphingolipid metabolism after HIF-PHI.

PLs are integral parts of the membrane and have important functional, structural, and metabolic roles (Liu, 2013). Recently, LC-MS/MS-based lipidomics has provided an opportunity to understand the pathological role of PLs and to develop new predictive biomarkers for distinct diseases.

Sphingolipids are an important class of lipid metabolites and have been confirmed as critical regulators of cardiovascular disease and cancer (Lemaitre, 2018). Abnormal sphingolipid metabolism is common in patients with uremia. Our previous study found that elevated levels of active sphingolipids are associated with a poor prognosis in end-stage renal disease dialysis (Tang, 2014b; Bai, 2020). Nete also reported that sphingomyelin and phosphatidylcholine species are associated with renal impairment and cause mortality in Type 1 diabetes (Tofte, 2019). In the present study, after Roxadustat treatment, the levels of most plasma sphingomyelin decreased to some extent, whereas only serum SM (d18:1/17:0) decreased significantly (*p* < 0.05).

Chen H. (2017a) reported a significant increase in the serum levels of phospholipids in patients with CKD, which was positively correlated with the level of serum triglycerides and inversely correlated with the levels of total cholesterol and eGFR (Chen H., 2017a). In the present study, we demonstrated that serum LysoPC (20:0), LysoPE (14:0), and PC (18:2) cis-dlpc levels were dramatically decreased after the HIF-PHI treatment. As it is reported that multiple PC, LPC, and LPE are correlated with cardiovascular morbidity (Ferreira-Divino, 2022; Jensen, 2022); thus, our findings that decreased phospholipids after the HIF-PHI treatment, might be suggesting a possible role in cardiovascular protection. However, further studies are required to confirm this hypothesis.

Ceramides are important precursors of other biological sphingolipids. Studies have shown that ceramide levels are increased in chronic kidney disease (CKD) patients (Mantovani, 2021) and are associated with cardiac and renal lipotoxicity (Savira, 2021). Interestingly, adipocyte HIF-2α deficiency exacerbates Western diet-induced atherosclerosis by increasing ceramide levels in adipose tissue, and activation of fatty HIF-2α by the HIF-PHI (Roxadustat) protects against atherosclerosis while simultaneously reducing fat, plasma ceramide, and plasma cholesterol levels (Zhang et al., 2019). In the present study, after the HIF-PHI administration, the levels of most of the plasma ceramides decreased to some extent but not significantly. These decreasing trends may suggest the potential role of the HIF pathway in sphingolipid metabolism; however, future studies with larger sample sizes are needed to confirm this.

One interesting finding in the present study was that decreased levels of phospholipids, lysophospholipids, and SM metabolism were correlated with the improvement of anemia after Roxadustat treatment.

However, the underlying mechanism remains unknown. Studies have shown that the life span of red blood cell (RBC) in patients with renal failure is significantly reduced, indicating that one of the reasons for renal anemia may be the reduction in the life span of RBCs (Vos, 2011; Sato, 2012; Ma, 2017; Li, 2019). In addition, it stabilizes HIF by inhibiting PHD, thereby promoting EPO secretion to improve renal anemia. A previous study also showed that hemodialysis (HD) patients treated with the HIF-PHI had significantly longer RBC longevity than those on rhuEPO therapy (Yang, 2021). Interestingly, both plasma and erythrocyte phospholipid metabolism have been linked to the RBC lifespan in previous studies (Grace, 2012; Dinkla, 2016). Further studies are needed to elucidate the link between phospholipids and RBC life after HIF-PHI treatment.

This study has several limitations. First, the sample size was relatively small, which reduced the statistical power of this study. Second, this is a real-world treatment cohort; although we tried our best to exclude the possible influence of other clinical and treatment factors, we cannot completely rule out the possible impact of some other factors that we did not include. Third, some patients were switched from ESA treatment to Roxadustat therapy, therefore, we could exclude the effect of withdraw of ESA treatment. Forth, despite independent relationship of change of hemoglobin and some of PLs and SMs were discovered after Roxadustat treatment in this study, we could not answer the underline mechanism. Therefore, further study using more advanced method to investigate the potential role of HIF-PHI in PLs and SMs metabolism is need in this area. Despite the above drawbacks our study is the first to investigate PLs and SMs metabolism after the HIF-PHI treatment in a real-world ESKD treatment cohort and may help understand the clinical effect of HIF-PHI in ESKD patients.

## Clinical significance

Elevated Phospholipids and sphingolipid (SM) metabolism have been connected with poor clinical status and adverse outcome of end-stage renal disease patients who underwent peritoneal dialysis (PD).

Hypoxia-inducible factor prolyl hydroxylase inhibitor (HIF-PHI) (Roxadustat) is a novel small-molecule oral drug used for the treatment of renal anemia. Several clinical studies have suggested that treatment by HIF-PHI is accompanied by a reduction in blood lipid levels. Study showed that atherosclerosis amelioration can be pharmacologically achieved in mice by activating adipose HIF-2α via the HIF-PHI (Roxadustat) possible by promoting ceramide catabolism.

This study demonstrated that after HIF-PHI administration, phospholipid and sphingolipid metabolism showed a decreasing trend. Decreased levels of phospholipids, lysophospholipids, and sphingolipid metabolism were correlated with the improvement of anemia after HIF-PHI (Roxadustat) treatment. Our findings that decreased phospholipids and sphingolipid metabolism after Roxadustat treatment, might be suggesting a possible protection role of HIF-PHI for PD patients besides correction of anemia. And provoke further investigation as clinically appropriate.

## Data Availability

The original contributions presented in the study are included in the article/Supplementary Material, further inquiries can be directed to the corresponding authors.
